# Multiscale higher-order molecular simplicial complex embedding for drug response prediction

**DOI:** 10.1093/bioinformatics/btag539

**Published:** 2026-07-22

**Authors:** Cong Shen, Guancen Lin, Chuan-Shen Hu, Jiawei Luo

**Affiliations:** State Key Laboratory of Mathematical Sciences, Academy of Mathematics and Systems Science, Chinese Academy of Sciences, Beijing, Beijing 100190, China; State Key Laboratory of Mathematical Sciences, Academy of Mathematics and Systems Science, Chinese Academy of Sciences, Beijing, Beijing 100190, China; Department of Applied Mathematics, National University of Kaohsiung, Kaohsiung 81148, Taiwan; College of Computer Science and Electronic Engineering, Hunan University, Changsha, Hunan 410082, China

## Abstract

**Motivation:**

Accurately predicting anticancer drug response is a central challenge in precision oncology. Existing computational methods, although valuable, often depend on pairwise molecular descriptors or limited graph-based encodings that cannot fully capture the complexity of molecular structures or their interactions with cellular states. These constraints hinder their robustness and generalization across diverse drugs and biological contexts, underscoring the need for more expressive frameworks.

**Results:**

To address this gap, we propose MolDr, a topological deep learning framework that represents molecules as multiscale simplicial complexes and propagates information across higher-order structures. By integrating these molecular representations with cellular profiles, MolDr unifies chemical topology and biological context within a single predictive model. Comprehensive experiments show that MolDr consistently outperforms or matches state-of-the-art baselines across multiple benchmarks. It achieves stronger accuracy and robustness on continuous drug response tasks, while also generalizing effectively to discrete classification settings. Moreover, sensitivity analysis confirms the benefit of incorporating multiple topological scales, further supporting the importance of higher-order representations. Together, these results demonstrate that MolDr delivers reliable performance across heterogeneous pharmacogenomic scenarios and highlight the promise of topological modeling for advancing drug response prediction.

**Availability:**

Source code freely available at https://github.com/CS-BIO/MolDr.

## 1 Introduction

Cancer remains one of the leading causes of mortality worldwide, and accurate drug response prediction (DRP) is a central problem in precision oncology (Vasan *et al.* 2019, Shen *et al.* 2026b). Predicting how tumors or cell lines respond to therapeutic compounds (e.g. IC_50_ or AUC) can guide personalized treatment, accelerate drug discovery, and improve understanding of resistance mechanisms (Iorio *et al.* 2016). However, traditional approaches such as high-throughput screening and clinical trials are costly, time-consuming, and limited in coverage, motivating the development of computational drug response prediction (DRP) methods based on large-scale pharmacogenomic data (Costello *et al.* 2014, Baptista *et al.* 2023).

Early DRP models relied on classical machine learning and handcrafted features, such as molecular fingerprints (e.g. ECFP, MACCS) combined with cell-line omics data, and applied methods like SVMs, random forests, or regression models (Hearst *et al.* 1998, Breiman 2001). While these approaches demonstrated feasibility, their limited structural expressiveness restricted generalization. With the rise of deep learning, graph neural networks (GNNs) have become the dominant paradigm, modeling molecules as graphs and learning representations via message passing (Liu *et al.* 2024, Shi *et al.* 2025). However, despite their success, these methods fundamentally rely on pairwise molecular graphs, where vertices represent atoms and edges represent chemical atom-pair interactions (e.g. chemical bonds). As a result, higher-order geometric relationships among multiple atoms are not explicitly modeled. Despite these advances, most GNN-based methods remain restricted to pairwise interactions and overlook higher-order molecular structures such as rings, cliques, and multi-body interactions, which play essential roles in determining molecular geometry, physicochemical properties, and biological activity (Li *et al.* 2022, Jiang *et al.* 2024). Consequently, important geometric and topological information may be lost, limiting model expressiveness and robustness.

To overcome this limitation, increasing attention has been devoted to higher-order graph learning. Hypergraph neural networks extend conventional graph representations by modeling group-wise interactions through hyperedges (Feng *et al.* 2019, Gao *et al.* 2022). Simplicial neural networks further provide a mathematically rigorous framework for representing multi-body interactions via simplicial complexes and enable message passing across simplices of different orders (Chen *et al.* 2022, Huang *et al.* 2024). More recently, topological molecular representation learning has demonstrated that geometric-topological structures extracted from molecular conformations can provide complementary molecular representations beyond conventional molecular graphs (Wasserman 2018, Shen *et al.* 2026a). Despite these advances, existing approaches have largely focused either on general higher-order graph learning or on molecular representation learning itself. Their integration into drug response prediction remains relatively limited, particularly with respect to jointly modeling multiscale molecular topology together with cellular biological context.

Throughout this work, we use *topological deep learning* as the overarching paradigm, referring to neural networks that explicitly exploit higher-order topological structures beyond ordinary graphs. Within this paradigm, simplicial complexes provide the mathematical representation of molecular topology, while higher-order propagation denotes the message-passing mechanism operating across simplices of different orders. These concepts provide the conceptual foundation of MolDr and motivate the proposed multiscale higher-order framework for drug response prediction.

Motivated by these challenges, we propose MolDr, a topology-aware drug response prediction framework that integrates multiscale molecular simplicial representations with cellular gene-expression profiles. We emphasize that the novelty of MolDr does not lie in the use of simplicial complexes alone, since higher-order graph learning and simplicial neural networks have been studied in related fields. Instead, MolDr contributes a task-oriented integration of three components for drug response prediction: (i) a Vietoris–Rips-based multiscale molecular construction that represents atoms, atom-pair interactions, and local three-atom configurations as 0-, 1-, and 2-simplices; (ii) a two-layer higher-order propagation mechanism that enables bidirectional information exchange between vertices and higher-order simplices; and (iii) a multiscale fusion strategy that combines molecular topological representations with cell-line expression embeddings for drug–cell response modeling. This design distinguishes MolDr from existing drug response prediction models that mainly rely on pairwise molecular graphs or generic graph encoders, and from general higher-order neural networks that are not specifically tailored to pharmacogenomic prediction.

## 2 Methods

In this section, we describe the proposed MolDr framework, including multiscale simplicial complex construction, higher-order information propagation, multiscale feature integration, and model optimization. For ease of reference, the main mathematical notations used throughout the proposed framework are summarized in Table S1, available as supplementary data at *Bioinformatics* online.

### 2.1 Multiscale topological representation for molecules

#### 2.1.1 Simplicial complex representation for molecules

A *simplicial complex* serves as a higher-dimensional generalization of a molecular graph, allowing for the explicit representation of atoms, atom-pair interactions, and their higher-order interactions. Roughly speaking, a simplicial complex consists of elementary geometric objects known as *simplices*, together with their face relationships, and organizes them into a combinatorial structure that captures the topology of the underlying space. Specifically, for a collection $S=\{v_{0},v_{1},\ldots ,v_{k}\}$ of k+1 affinely independent points in $R^{d}$ (d≥k), the associated *k*-simplex σ, depending on the set *S*, is defined as the convex hull of these points. That is,


$$\begin{array}{c} \sigma =\mathrm{conv}(S)=\mathrm{conv}(v_{0},v_{1},\ldots ,v_{k})\\ =\left\{\sum_{i=0}^{k}\lambda_{i}v_{i}\;|\;\lambda_{i}\geq 0,\;\sum_{i=0}^{k}\lambda_{i}=1\right\}. \end{array}$$

Note that a simplex σ is uniquely determined, up to its vertices $v_{0},v_{1},\ldots ,v_{k}$, by convex combinations of these vertices. The dimension of σ is therefore defined as dim(σ) :=|S|−1=k. In particular, a *k*-simplex is denoted by $\sigma^{k}$ when its dimension is to be emphasized.

For a given *k*-simplex $\sigma =\mathrm{conv}(v_{0},v_{1},\ldots ,v_{k})$ with $S=\{v_{0},v_{1},\ldots ,v_{k}\}$ being an (ordered) affinely independent set in $R^{d}$, and for an index i∈{0,1,…,k}, the *i*th (k−1)  *-face* of σ is defined as the (k−1)-simplex generated by the set $S\setminus \{v_{i}\}$; that is,


$$\tau_{i}=\mathrm{conv}(v_{0},\ldots ,\hat{v_{i}},\ldots ,v_{k}),$$

Where the notation $\hat{v_{i}}$ indicates omission of the vertex $v_{i}$. More generally, an *l*-*face* of a *k*-simplex σ=conv(S), generated by an affinely independent set *S* with |S|=k+1, where 0≤l≤k−1, is the *l*-simplex conv(T) generated by a subset T⊆S satisfying |T|=l+1. In particular, the boundary of a *k*-simplex $\sigma =\mathrm{conv}(v_{0},v_{1},\ldots ,v_{k})$ is the set


$$\partial (\sigma )=\overset{k}{\underset{i=0}{\cup }}\;\mathrm{conv}(v_{0},\ldots ,\hat{v_{i}},\ldots ,v_{k})$$

As a union of all the (k−1)-faces of σ. In other words, the boundary of a set of k+1 affinely independent points in R^*d*^ encodes the topological relations between the entire simplex spanned by these points and its (k−1)-dimensional faces, each corresponding to a subset of *k* points.

#### 2.1.2 Filtration-based multiscale representation

To capture molecular structures across different geometric resolutions, a multiscale mathematical procedure based on interatomic distances, known as a *topological filtration*, is employed. In the proposed approach, the Vietoris–Rips construction is employed to encode molecular structures. We adopt the Vietoris–Rips filtration because it naturally constructs simplicial complexes directly from pairwise interatomic distances without requiring additional geometric preprocessing. Compared with geometric filtrations such as the Alpha complex, the Vietoris–Rips construction provides a straightforward multiscale representation that is well suited for higher-order message passing while remaining computationally efficient for large-scale pharmacogenomic datasets. Specifically, for a molecular system with a set *X* of atomic coordinates in R^3^ and an increasing sequence of nonnegative real numbers $r_{1}<r_{2}<\cdots <r_{s}$, the associated *Vietoris–Rips filtration* is defined as a sequence of simplicial complexes ${\left\{{\mathrm{Rips}}_{r_{i}}(X)\right\}}_{i=1}^{s}$ parameterized by the cutoff values ${\left\{r_{i}\right\}}_{i=1}^{s}$, such that


(1) $${\mathrm{Rips}}_{r_{1}}(X)\subseteq {\mathrm{Rips}}_{r_{2}}(X)\subseteq \cdots \subseteq {\mathrm{Rips}}_{r_{s}}(X),$$

Where each complex ${\mathrm{Rips}}_{r_{i}}(X)$ is constructed from the atomic coordinates as its vertices (i.e. 0-simplices). Formally, for a point set $X=\{x_{1},x_{2},\ldots ,x_{n}\}\subseteq {\mathbb{R}}^{3}$, we define Simp(X) as the set of all simplices in R^3^ whose vertices are points of *X*. The Vietoris–Rips complex at scale *r* is then defined as the simplicial complex


$${\mathrm{Rips}}_{r}(X)=\{\sigma \in \mathrm{Simp}(X)|\|x_{i}-x_{j}\|\leq r\;\text{for}\;\text{all}\;x_{i},x_{j}\in \sigma \},$$

So that a simplex $\sigma \subseteq R^{3}$ is included as an element in ${\mathrm{Rips}}_{r}(X)$ whenever all pairwise distances between its vertices are at most *r*.

This process yields a family of nested complexes as in Equation (1), where increasing the cutoff value systematically introduces higher-order simplices. Motivated by the chemical intuition that bond lengths determine feasible molecular interactions, we select cutoff values


r∈{2.0,2.5,3.0,3.5,4.0} Å.

In this way, the proposed model captures both local interactions governed by short bonds and increasingly global structural dependencies that emerge at longer distances.

From a chemical perspective, 2-simplices encode local three-atom configurations rather than isolated pairwise atom interactions. Such configurations naturally arise in molecular motifs including bond angles, aromatic ring fragments, and locally coordinated atomic arrangements, all of which contribute to molecular geometry and influence physicochemical properties. Although a 2-simplex does not explicitly represent a specific chemical interaction, it provides a compact higher-order structural unit that complements conventional bond-based representations by capturing the geometric context surrounding neighboring atoms. Therefore, incorporating 2-simplices allows MolDr to model molecular organization beyond pairwise connectivity while maintaining a clear geometric interpretation.

#### 2.1.3 Adjacency relations in simplicial complexes

To facilitate information propagation within the simplicial complex, adjacency relations are defined across simplices of different dimensions. In our model, the neighborhood of a vertex comprises three types: other 0-simplices that form a 1-simplex with the vertex, incident 1-simplices containing the vertex, and incident 2-simplices containing the vertex. Formally, for the Vietoris–Rips complex ${\mathrm{Rips}}_{r}(X)$ at cutoff *r*, the adjacency matrix $A_{r,k}=\{A_{r,k}(i,j)\}$ for k≥0 is defined as follows.

For 0-simplices, two vertices are adjacent if and only if they span a common 1-simplex:


$$A_{r,0}(i,j)=\{\begin{array}{cc} 1, & \text{if}\;\sigma_{i}^{0},\sigma_{j}^{0}\subseteq \sigma^{1},\;i\neq j,\\ 0, & \text{otherwise}. \end{array}$$

For k≥1, a *k*-simplex $\sigma_{i}^{k}$ is adjacent to another *k*-simplex $\sigma_{j}^{k}$ if they share a common (k−1)-face:


$$A_{r,k}(i,j)=\left\{ \begin{array}{cc} 1, & \text{if}\;\sigma_{i}^{k}\cap \sigma_{j}^{k}\in {\mathrm{Rips}}_{r}(X),\;dim(\sigma_{i}^{k}\cap \sigma_{j}^{k})=k,\;i\neq j,\\ 0, & \text{otherwise}. \end{array}\right.$$

In particular, this definition ensures that two 1-simplices are adjacent if they share a common incident vertex, and two 2-simplices are adjacent if they share a common incident 1-simplex.

To normalize the adjacency matrices, we introduce diagonal degree matrices. The *upper degree* of a *k*-simplex $\sigma_{i}^{k}$, denoted by $\deg_{U}(\sigma_{i}^{k})$, is defined as the number of (k+1)-simplices for which $\sigma_{i}^{k}$ is a face. The corresponding diagonal matrix is given by


$$D_{r,k}^{U}(i,j)=\{\begin{array}{cc} \deg_{U}(\sigma_{i}^{k}), & \text{if}\;i=j,\\ 0, & \text{otherwise}. \end{array}$$

Similarly, the *lower degree* of a *k*-simplex $\sigma_{i}^{k}$, denoted $\deg_{L}(\sigma_{i}^{k})$, is the number of (k−1)-faces that it contains. The associated diagonal matrix is


$$D_{r,k}^{L}(i,j)=\{\begin{array}{cc} \deg_{L}(\sigma_{i}^{k}), & \text{if}\;i=j,\\ 0, & \text{otherwise}. \end{array}$$

Collectively, $A_{r,k}$, $D_{r,k}^{U}$, and $D_{r,k}^{L}$ formally characterize the adjacency and degree structure within the simplicial complex, enabling us to rigorously define neighborhood relations across 0-, 1-, and 2-simplices for subsequent message passing.

### 2.2 Multiscale higher-order information propagation on simplicial complexes

#### 2.2.1 First-layer propagation: vertex-centric aggregation

The first propagation layer adopts a vertex-centric perspective. Each vertex feature is initially updated using standard graph convolution over the 1-skeleton of the simplicial complex:


$$X^{\left(1\right)}=\sigma (\hat{A}X^{\left(0\right)}W_{v}^{\left(1\right)}),$$

where $\hat{A}$ denotes the normalized adjacency matrix, $W_{v}^{\left(1\right)}$ is a learnable parameter matrix, and σ(·) is a nonlinear activation function. Beyond the conventional vertex–vertex interactions, higher-order neighborhood information is also aggregated. For a vertex *v*, the contribution from its incident 1-simplices is given by


$$x^{\left(e\right)}(v)=\frac{1}{\left|E(v)\right|}\sum_{e\in E(v)}E_{e}^{\left(0\right)},$$

While the contribution from its incident 2-simplices is expressed as


$$x^{\left(t\right)}(v)=\frac{1}{\left|T(v)\right|}\sum_{\tau \in T(v)}T_{\tau }^{\left(0\right)}.$$

These components are concatenated with the updated vertex embedding and passed through an MLP:


$$H_{v}^{\left(1\right)}=\psi_{v}^{\left(1\right)}([X_{v}^{\left(1\right)}\|x^{\left(e\right)}(v)\|x^{\left(t\right)}(v)]).$$

This operation establishes the initial integration of local vertex information with the signals from higher-order simplices, providing each vertex with a representation that reflects both pairwise and higher-order molecular structures.

#### 2.2.2 Second-layer propagation: higher-order feedback

The second layer introduces a feedback mechanism, in which updated vertex embeddings are used to refine the representations of 1- and 2-simplices, and these higher-order embeddings are then fed back to further enhance the vertex features. First, vertex embeddings are updated by another round of graph convolution:


$${\tilde{H}}^{\left(1\right)}=\sigma (\hat{A}H^{\left(1\right)}W_{v}^{\left(2\right)}).$$

For each 1-simplex e=(u,v), the updated features of its endpoints are concatenated with its original feature:


$$E_{e}^{\left(1\right)}=\psi_{e}([{\tilde{H}}_{u}^{\left(1\right)}\|{\tilde{H}}_{v}^{\left(1\right)}\|E_{e}^{\left(0\right)}]).$$

Similarly, for each 2-simplex τ=(u,v,w), the updated features of its three vertices are combined with its initial feature:


$$T_{\tau }^{\left(1\right)}=\psi_{t}([{\tilde{H}}_{u}^{\left(1\right)}\|{\tilde{H}}_{v}^{\left(1\right)}\|{\tilde{H}}_{w}^{\left(1\right)}\|T_{\tau }^{\left(0\right)}]).$$

The enriched simplex features are then propagated back to the vertices by averaging over their incident 1- and 2-simplices:


$$h^{\left(e\right)}(v)=\frac{1}{\left|E(v)\right|}\sum_{e\in E(v)}E_{e}^{\left(1\right)},\;h^{\left(t\right)}(v)=\frac{1}{\left|T(v)\right|}\sum_{\tau \in T(v)}T_{\tau }^{\left(1\right)},$$

And concatenating them with the updated vertex feature:


$$H_{v}^{\left(2\right)}=\psi_{v}^{\left(2\right)}([{\tilde{H}}_{u}^{\left(1\right)}\|h_{\;}^{\left(e\right)}(v)\|h_{\;}^{\left(t\right)}(v)]).$$

Through this two-stage design, the framework establishes a complete information loop: vertices first aggregate signals from neighboring simplices, and the updated higher-order simplices then feed back to refine vertex representations. This process enables coherent integration of local and higher-order structural information. The initialization of simplex features is provided in the Supplementary Materials, available as supplementary data at *Bioinformatics* online.

### 2.3 Readout and multiscale integration

After the two-layer message passing, each vertex is associated with a refined embedding $H_{v}^{\left(2\right)}$. To obtain a molecular-level representation, we apply a permutation-invariant readout function. Specifically, for a simplicial complex constructed under cutoff α, we compute


$$z_{\alpha }=\rho_{V}(\{H_{v}^{\left(2\right)}:v\in V\})\in R^{d_{z}},$$

where $\rho_{V}(\cdot )$ denotes a pooling operation such as mean, sum, or attention-based aggregation.

To integrate information across multiple filtration scales, embeddings from different cutoffs are concatenated. Let $\left\{r_{1},r_{2},\ldots ,r_{S}\right\}$ denote the set of cutoff values, then the final molecular representation is given by


$$z_{\text{mol}}=\psi_{\text{mol}}([z_{r_{1}}\|z_{r_{2}}\|\cdots \|z_{r_{S}}]),$$

where $\psi_{\text{mol}}(\cdot )$ is an MLP for multiscale fusion. The resulting embedding captures structural information across different geometric scales.

### 2.4 Cell-line embedding and fusion with molecular features

While the molecular representation encodes structural and chemical information of the drug, the cellular context is equally important for determining drug response. To represent each cell line, we take its gene expression profile $g\in R^{p}$, normalize it, and map it into a lower-dimensional embedding space using an MLP:


$$z_{\text{cell}}=\psi_{\text{cell}}(g)\in R^{d_{c}}.$$

This cell-line embedding captures the intrinsic biological state of the cell, including transcriptional programs and pathway activities that influence drug sensitivity.

The final prediction is based on the joint representation of the drug and the cell line. We concatenate the molecular embedding with the cell-line embedding,


$$z=[z_{\text{mol}}\|z_{\text{cell}}],$$

And apply a prediction head implemented as another MLP to produce a scalar value representing the drug response:


$$\hat{y}=\psi_{\text{pred}}(z).$$

Here, $\hat{y}$ denotes the predicted drug sensitivity score for the given drug–cell line pair.

### 2.5 Optimization objective

**Figure 1:**
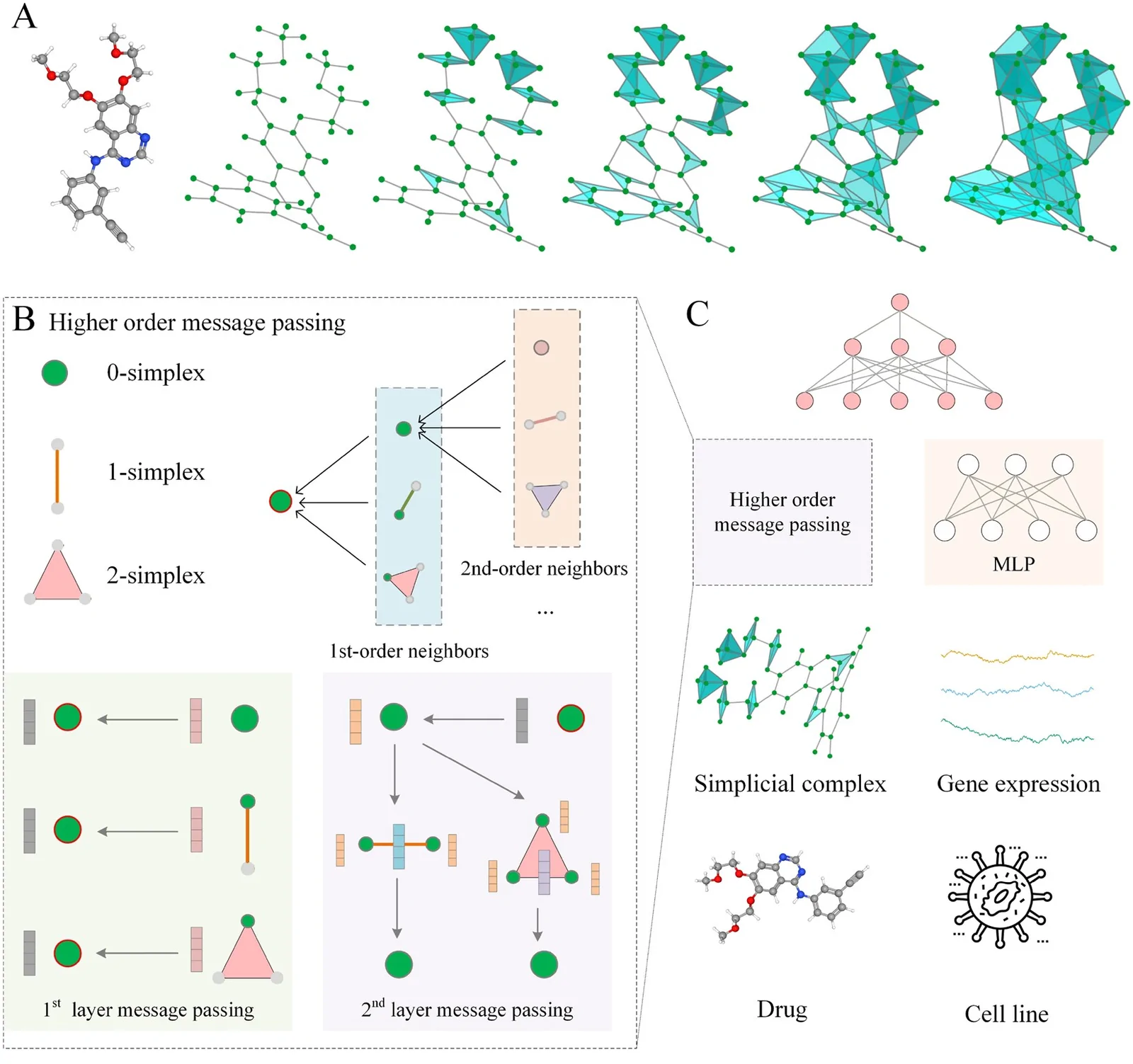
The overall framework of MolDr. (A) Construction of multiscale simplicial complexes, where 0-, 1-, and 2-simplices correspond to atoms, atom-pair interactions, and local three-atom configurations, respectively. (B) Higher-order message passing on simplicial complexes. (C) Overall predictive framework.

The model is trained in an end-to-end manner using a regression objective. Specifically, we adopt the mean squared error (MSE) between predicted responses ${\hat{y}}_{i}$ and ground-truth responses $y_{i}$:


$$L=\frac{1}{N}\sum_{i=1}^{N}{\left({\hat{y}}_{i}-y_{i}\right)}^{2},$$

where *N* is the number of drug–cell line pairs. By optimizing this loss function, the framework learns to jointly capture structural properties of molecules and biological states of cell lines, thereby improving predictive performance on drug response tasks. The overall framework of MolDr is illustrated in Fig. 1.

### 2.6 Complexity analysis

Let $N_{0}$, $N_{1}$, and $N_{2}$ denote the numbers of 0-, 1-, and 2-simplices, respectively. The complexity of the proposed framework is therefore $O\left(\mathrm{SLd}(N_{0}+N_{1}+N_{2})\right)$, where *S* denotes the number of filtration scales and *L* the number of propagation layers. Since both *S* and *L* are fixed constants in MolDr, the complexity is linear with respect to the size of the simplicial complex.

## 3 Results

### 3.1 Overall performance comparison

To rigorously assess MolDr, we conduct experiments on six benchmarks, TGSA (Zhu *et al.* 2022), GDSC1 (Yang *et al.* 2012), GDSC2 (Yang *et al.* 2012), CCLE (Barretina *et al.* 2012), CTRP1 (Basu *et al.* 2013), and CTRP2 (Basu *et al.* 2013). Detailed statistics of the six benchmark datasets are provided in Table S2, available as supplementary data at *Bioinformatics* online. We compare MolDr with fifteen representative baselines spanning four categories: (i) Domain-specific drug response prediction (DRP) methods: SubCDR (Liu and Zhang 2023), GraphCDR (Liu *et al.* 2022), PANCDR (Kim *et al.* 2024), MSDRP (Zhao *et al.* 2023), and MultiDRP (Wang *et al.* 2024). (ii) General-purpose graph neural networks (GNNs): SSGC (Zhu and Koniusz 2021), GraphGPS (Rampášek *et al.* 2022), EGC (Tailor *et al.* 2021), ARMA (Bianchi *et al.* 2022), and A-DGN (Gravina *et al.* 2023). (iii) Higher-order and topology-aware graph learning methods: HGNN (Feng *et al.* 2019),  HGNN^+^ (Gao *et al.* 2022), BScNets (Chen *et al.* 2022), and HiGCN (Huang *et al.* 2024). (iv) A pretrained molecular foundation model: MolGT (Chen *et al.* 2025). The complete results are listed in Table 1. Detailed descriptions of the compared methods, data preprocessing, and experimental settings are provided in the Supplementary Materials, available as supplementary data at *Bioinformatics* online.

**Table 1:** Performance comparison of different methods across six benchmark datasets.

Metric	Dataset	SSGC	GraphGPS	EGC	ARMA	A-DGN	HGNN	**HGNN^+^**	BScNets	HiGCN	MolGT	SubCDR	GraphCDR	PANCDR	MSDRP	MultiDRP	MolDr
MAE	TGSA	0.819	0.753	0.784	0.784	0.771	0.906	1.092	0.894	1.281	1.090	0.844	1.130	0.980	0.741	0.735	**0.727**
	GDSC1	0.819	0.738	0.749	0.741	0.810	0.907	1.020	0.861	0.862	1.044	0.757	0.919	0.834	0.764	0.775	**0.722**
	GDSC2	0.797	0.727	0.759	0.746	0.782	0.913	1.068	0.865	0.859	1.041	0.778	0.941	0.776	0.743	0.752	**0.708**
	CCLE	0.488	0.479	0.481	0.477	0.489	0.501	0.543	0.470	0.473	0.784	0.486	0.616	0.493	0.474	**0.469**	**0.469**
	CTRP1	0.758	0.743	0.766	0.775	0.776	0.941	1.177	0.888	0.964	1.199	0.840	0.904	0.958	**0.740**	0.775	0.751
	CTRP2	0.891	0.808	0.831	0.821	0.909	0.948	1.079	0.976	1.034	1.316	0.922	1.222	0.817	0.818	0.877	**0.795**
PCC	TGSA	0.924	0.935	0.930	0.930	0.931	0.906	0.863	0.907	0.901	0.871	0.920	0.896	0.897	0.934	0.935	**0.938**
	GDSC1	0.911	0.926	0.927	0.926	0.913	0.891	0.860	0.901	0.901	0.867	0.926	0.884	0.908	0.923	0.921	**0.929**
	GDSC2	0.929	0.940	0.935	0.937	0.932	0.904	0.869	0.912	0.916	0.887	0.933	0.906	0.935	0.937	0.935	**0.942**
	CCLE	0.869	0.876	0.874	0.876	0.869	0.852	0.815	0.873	0.872	0.431	0.861	0.823	0.864	0.872	0.876	**0.878**
	CTRP1	0.803	0.816	0.803	0.798	0.803	0.699	0.503	0.733	0.713	0.468	0.773	0.726	0.708	**0.819**	0.796	0.801
	CTRP2	0.880	0.903	0.896	0.898	0.877	0.861	0.818	0.850	0.839	0.711	0.872	0.756	0.898	0.895	0.880	**0.903**
*R* ^2^	TGSA	0.852	0.874	0.864	0.864	0.865	0.821	0.745	0.821	0.800	0.771	0.841	0.746	0.789	0.871	0.875	**0.880**
	GDSC1	0.828	0.858	0.858	0.857	0.833	0.789	0.736	0.810	0.812	0.695	0.855	0.781	0.822	0.851	0.848	**0.863**
	GDSC2	0.864	0.884	0.873	0.878	0.868	0.817	0.755	0.832	0.836	0.792	0.867	0.808	0.872	0.877	0.873	**0.887**
	CCLE	0.755	0.768	0.764	0.767	0.754	0.732	0.668	0.761	0.763	0.210	0.734	0.625	0.745	0.760	0.767	**0.768**
	CTRP1	0.642	0.662	0.640	0.629	0.640	0.486	0.258	0.536	0.485	0.215	0.590	0.522	0.482	**0.670**	0.632	0.635
	CTRP2	0.772	0.814	0.800	0.805	0.769	0.740	0.673	0.720	0.727	0.483	0.757	0.569	0.806	0.801	0.771	**0.814**

The best results are highlighted in bold.

Overall, MolDr shows the consistently strong performance across the six benchmark datasets. It achieves the best MAE (tied with MultiDRP) on TGSA, GDSC1, GDSC2, CCLE and CTRP2, and remains competitive on CTRP1. For PCC and *R*^2^, MolDr also ranks first on most datasets, indicating consistent improvements across different evaluation metrics. On GDSC1, compared with the strongest competing baseline MultiDRP, MolDr reduces MAE from 0.775 to 0.722, while improving PCC from 0.921 to 0.929 and *R*^2^ from 0.848 to 0.863. On GDSC2, MolDr achieves the best overall performance (MAE/PCC/*R*^2^ = 0.708/0.942/0.887), outperforming all competing methods. On CTRP2, MolDr consistently outperforms the strongest competing methods across all three metrics. In contrast, on CTRP1, MolDr performs comparably to the top baseline MSDRP, with only a marginal difference in three evaluation metrics. Consistent with these results, t-SNE visualization (Fig. S1, available as supplementary data at *Bioinformatics* online) shows that MolDr learns more structured and discriminative drug–cell embeddings than the initial features. These results demonstrate that the performance advantage of MolDr generalizes across datasets and evaluation metrics.

### 3.2 Ablation rationale and design

To evaluate the contribution of higher-order topology, we first perform simplex-order ablations by progressively removing different simplex types (Table 2). The 0-,1-simplex and 0-,2-simplex variants retain only bond-level and triangle-level higher-order propagation, respectively, whereas the full MolDr incorporates both 1- and 2-simplices. All other components remain unchanged.

**Table 2:** Ablation over simplex orders on six datasets.

Dataset	Methods	MAE	PCC	*R* ^2^	Dataset	Methods	MAE	PCC	*R* ^2^
TGSA	0 -simplex	0.738	0.921	0.870	GDSC1	0 -simplex	0.733	0.911	0.846
	0 -, 1 -simplex	0.735	0.929	0.875		0 -, 1 -simplex	0.726	0.920	0.854
	0 -, 2 -simplex	0.736	0.929	0.874		0 -, 2 -simplex	0.731	0.919	0.853
	MolDr	0.727	0.938	0.880		MolDr	0.722	0.929	0.863
GDSC2	0 -simplex	0.721	0.934	0.881	CCLE	0 -simplex	0.480	0.861	0.748
	0 -, 1 -simplex	0.713	0.940	0.883		0 -, 1 -simplex	0.472	0.868	0.752
	0 -, 2 -simplex	0.715	0.941	0.886		0 -, 2 -simplex	0.473	0.868	0.752
	MolDr	0.708	0.942	0.887		MolDr	0.469	0.878	0.768
CTRP1	0 -simplex	0.784	0.783	0.605	CTRP2	0 -simplex	0.794	0.901	0.811
	0 -, 1 -simplex	0.774	0.785	0.614		0 -, 1 -simplex	0.793	0.902	0.813
	0 -, 2 -simplex	0.782	0.784	0.612		0 -, 2 -simplex	0.795	0.902	0.813
	MolDr	0.751	0.801	0.635		MolDr	0.795	0.903	0.814

Lower MAE and higher PCC/*R*^2^ indicate better performance.

MolDr consistently outperforms all simplex-order variants, demonstrating the benefit of higher-order topology. For example, on GDSC2, MAE decreases from 0.721 to 0.708 together with improvements in PCC and *R*^2^, while similar trends are observed on TGSA and CCLE. Comparing the two variants further shows that 1-simplices mainly improve PCC and *R*^2^, whereas 2-simplices further reduce MAE, suggesting that bond-level interactions capture the dominant structural dependencies while three-atom configurations provide complementary geometric information.

We further evaluate three additional architectural components, including propagation depth (Table S3, available as supplementary data at *Bioinformatics* online), the cell-line embedding module (Table S4, available as supplementary data at *Bioinformatics* online), and the multiscale fusion strategy (Table S5, available as supplementary data at *Bioinformatics* online). Across all six datasets, the proposed two-layer propagation consistently outperforms shallower or deeper variants, the MLP-based cell embedding provides clear gains over linear or removed embeddings, and the proposed concatenation + MLP fusion achieves the most consistent overall performance. Together, these results confirm that each component contributes to the final performance of MolDr.

### 3.3 Cold-start generalization analysis

To further evaluate the generalization ability of MolDr, we conducted cold-start experiments under two challenging settings: cell-line cold-start and drug cold-start. In the cell-line cold-start setting, cell lines in the test set were not observed during training, while in the drug cold-start setting, test drugs were excluded from the training set.

As shown in Table S6, available as supplementary data at *Bioinformatics* online, MolDr consistently outperforms MultiDRP across all six datasets under both cold-start settings. In the cell-line cold-start setting, MolDr achieves lower MAE and higher PCC/*R*^2^ on every dataset, indicating that the learned molecular-topological representation can generalize to unseen cellular contexts. In the more challenging drug cold-start setting, performance decreases for both methods, but MolDr still maintains clear advantages across all datasets. This suggests that the multiscale higher-order simplicial representation provides more transferable molecular features for unseen drugs.

### 3.4 Robustness to noisy cell-line expression

To assess robustness to noise in cellular profiles, we performed a stress test in which the gene-expression matrix was randomly corrupted. Specifically, for each dataset, 20% of the entries were randomly replaced with values drawn from the same normalized feature space. The same perturbation mask was applied to all methods, while keeping the original training and evaluation protocols unchanged. Performance was evaluated using MAE (lower is better), PCC, and *R*^2^ (higher are better), as summarized in Fig. S2 and Table S7, available as supplementary data at *Bioinformatics* online.

Overall, MolDr maintains the best or highly competitive performance across most datasets and metrics under this 20% corruption setting. It achieves the lowest MAE on TGSA, GDSC1, GDSC2, CCLE, and CTRP2, and attains the highest PCC and *R*^2^ on the majority of datasets. These results indicate that MolDr is less sensitive to noise in cell-line expression and can preserve both predictive accuracy and correlation structure under substantial perturbations. A slight exception is observed on CTRP1, where several baselines achieve marginally better results. This suggests that CTRP1 may rely more heavily on cell-line expression features, making it more sensitive to corruption. Nevertheless, MolDr remains competitive in this setting.

### 3.5 Cancer-specific versus pan-cancer drug response prediction

To evaluate the robustness of MolDr under different cancer-type distributions, we compared its performance in cancer-specific and pan-cancer settings on the TGSA dataset, considering three representative cancer types: BRCA, LUAD, and COREAD. In the cancer-specific setting, models are trained and tested within the same cancer type, whereas in the pan-cancer setting, the target cancer type is used only for testing and all other types are used for training and validation.

As shown in Table S8, available as supplementary data at *Bioinformatics* online, MolDr consistently outperforms MultiDRP across all cancer types in both settings, achieving lower MAE and higher PCC/$R^{2}$. Although performance generally degrades in the pan-cancer setting due to distribution shifts, MolDr maintains clear advantages, indicating its robustness. Overall, these results suggest that MolDr effectively captures structural signals that generalize across cancer types, supporting its applicability in both cancer-specific prediction and cross-cancer generalization.

### 3.6 Molecular construction analysis

Since MolDr constructs Vietoris–Rips complexes directly from molecular three-dimensional coordinates, we first investigate whether different molecular conformer generation strategies influence prediction performance. We compare four representative protocols, including Random embedding, ETKDG, ETKDG + MMFF optimization, and a lowest-energy multi-conformer strategy. To isolate the effect of conformer generation from multiscale fusion, this analysis is conducted using a single filtration scale (2.0 Å), and the results are summarized in Table S9, available as supplementary data at *Bioinformatics* online. Overall, MolDr exhibits highly consistent performance across different conformer generation strategies, indicating that the proposed framework is not overly sensitive to the choice of molecular conformers. Among them, ETKDG + MMFF achieves the best or near-best performance on most datasets, suggesting that force-field refinement provides more informative geometric structures for simplicial complex construction. Therefore, ETKDG + MMFF is adopted as the default conformer generation strategy throughout this work.

We next evaluate the influence of conformer uncertainty. Specifically, 20 conformers are generated for each of 100 randomly selected molecules using ETKDG followed by MMFF/UFF optimization, and Vietoris–Rips complexes are independently constructed for each conformer. The resulting geometric-topological structures are assessed using geometry similarity together with the coefficients of variation (CVs) of edge counts, triangle counts, mean edge lengths, and mean triangle areas (Table S10, available as supplementary data at *Bioinformatics* online). MolDr exhibits consistently high geometry similarity across all six datasets, while all structural and geometric statistics show only minimal variation. These results demonstrate that the higher-order molecular representations remain highly consistent across reasonable molecular conformers, confirming that MolDr is insensitive to conformer uncertainty.

Finally, we investigate the sensitivity of MolDr to filtration cutoff selection. Besides the comparison of different multiscale settings in Table 3, Fig. S3 and Table S11, available as supplementary data at *Bioinformatics* online further evaluate four shifted cutoff windows. These findings indicate that MolDr is not overly sensitive to the absolute cutoff values and that the proposed multiscale higher-order representation remains stable across a reasonable range of filtration scales. Accordingly, the cutoff set {2.0, 2.5, 3.0, 3.5} Å is adopted as the default configuration throughout this work.

**Table 3:** Performance of MolDr under different multiscale settings on six datasets. Here, 1-scale uses only the 2.0 Å cutoff; 2-scale uses the 2.0 Å and 2.5 Å cutoffs; 3-scale further includes the 3.0 Å cutoff; and 4-scale further includes the 3.5 Å cutoff.

Dataset	Scales	MAE	PCC	*R* ^2^	Dataset	Scales	MAE	PCC	*R* ^2^
TGSA	1-scale	0.730	0.937	0.878	GDSC1	1-scale	0.726	0.928	0.860
	2-scale	**0.727**	**0.938**	**0.880**		2-scale	0.724	0.929	0.862
	3-scale	0.730	0.938	0.879		3-scale	**0.722**	**0.929**	**0.863**
	4-scale	0.729	0.938	0.878		4-scale	0.722	0.929	0.863
GDSC2	1-scale	0.717	0.940	0.885	CCLE	1-scale	0.475	0.861	0.753
	2-scale	0.716	0.941	0.885		2-scale	0.474	0.865	0.748
	3-scale	**0.708**	**0.942**	**0.887**		3-scale	0.477	0.863	0.744
	4-scale	0.713	0.942	0.887		4-scale	**0.469**	**0.878**	**0.768**
CTRP1	1-scale	0.756	0.800	0.626	CTRP2	1-scale	0.798	0.901	0.811
	2-scale	0.756	0.800	0.631		2-scale	**0.795**	**0.903**	**0.814**
	3-scale	**0.751**	**0.801**	**0.635**		3-scale	0.798	0.902	0.812
	4-scale	0.755	0.797	0.624		4-scale	0.801	0.901	0.810

Lower MAE and higher PCC/*R*^2^ indicate better performance. Best results in each row are in **bold**.

### 3.7 Performance on discrete drug response datasets

To evaluate generalizability, we extend MolDr to binary drug response prediction using TCGA (Weinstein *et al.* 2013) and GDSC (Iorio *et al.* 2016), where responses are discrete rather than continuous. We follow the same 8:1:1 data split and adjust the learning objective from mean squared error to cross-entropy loss, evaluating performance using AUC, accuracy, precision, and F1-score. PANCDR (Kim *et al.* 2024) and DeepCDR (Liu *et al.* 2020) are used as baselines.

As shown in Fig. S4, available as supplementary data at *Bioinformatics* online, MolDr consistently outperforms baselines on TCGA, achieving higher ROC curves, and remains competitive on GDSC. It also maintains strong performance across accuracy, precision, and F1-score, often surpassing competing methods. These results indicate that MolDr effectively generalizes from regression to classification tasks. We further analyze parameter sensitivity with respect to the number of filtration intervals. Performance improves with additional scales, with the best results achieved using all four intervals, highlighting the importance of multiscale structural information and the robustness of MolDr.

## 4 Discussion and conclusion

In this work, we proposed MolDr, a multiscale higher-order topological deep learning framework for drug response prediction. By representing molecules as simplicial complexes and enabling information propagation across different simplex orders, MolDr captures structural information beyond conventional molecular graphs. Extensive experiments on six pharmacogenomic benchmarks demonstrate that MolDr consistently achieves competitive or state-of-the-art performance under multiple evaluation settings.

Beyond the benchmark improvements, our findings also have potential implications for precision oncology. Accurate computational prediction of drug response can help prioritize candidate therapies before costly experimental validation, thereby improving the efficiency of pharmacogenomic screening. By jointly modeling higher-order molecular topology and cellular gene expression, MolDr provides a richer representation of drug–cell interactions that may better capture subtle structural determinants of therapeutic response. As larger pharmacogenomic resources and patient-derived datasets continue to emerge, topology-aware molecular representations such as MolDr may become valuable computational components for future precision oncology research. Future work will investigate the integration of additional omics modalities, such as mutation, methylation, and chromatin accessibility profiles, together with patient-derived datasets, to further evaluate the clinical applicability and generalizability of the proposed framework.

## Supplementary Material

btag539_Supplementary_Data

## Data Availability

The datasets, preprocessing scripts and source code underlying this article are available in the MolDr repository at https://github.com/CS-BIO/MolDr.
