# Supplementary material for: Multiscale higher-order molecular simplicial complex embedding for drug response prediction
Source: Bioinformatics. 2026 Jul 22;42(8):btag539. doi: 10.1093/bioinformatics/btag539 (PMC13433265; doi:10.1093/bioinformatics/btag539)
Supplement: Supplementary file 1 [file btag539_supplementary_data.pdf]

*Journal Title Here*, 2022, pp. 1–10

doi: DOI HERE
Advance Access Publication Date: Day Month Year
Paper

PAPER

# Supplementary Materials: Multiscale Higher-Order Molecular Simplicial Complex Embedding for Drug Response Prediction

Cong Shen,[1,4] Guancen Lin,[1,4] Chuan-Shen Hu[2,*] and Jiawei Luo[3,*]

[1]State Key Laboratory of Mathematical Sciences, Academy of Mathematics and Systems Science, Chinese Academy of Sciences, Beijing, 100190, Beijing, China, [2]Department of Applied Mathematics, National University of Kaohsiung, 81148, Kaohsiung, Taiwan, [3]College of Computer Science and Electronic Engineering, Hunan University, Changsha, 410082, Hunan, China and [4]These authors contributed equally to this work.

[*]Corresponding author. chuanshenhu1@nuk.edu.tw, luojiawei@hnu.edu.cn

## Abstract

**Motivation:** Accurately predicting anticancer drug response is a central challenge in precision oncology. Existing computational methods, although valuable, often depend on pairwise molecular descriptors or limited graph-based encodings that cannot fully capture the complexity of molecular structures or their interactions with cellular states. These constraints hinder their robustness and generalization across diverse drugs and biological contexts, underscoring the need for more expressive frameworks.

**Results:** To address this gap, we propose MolDr, a topological deep learning framework that represents molecules as multiscale simplicial complexes and propagates information across higher-order structures. By integrating these molecular representations with cellular profiles, MolDr unifies chemical topology and biological context within a single predictive model. Comprehensive experiments show that MolDr consistently outperforms or matches state-of-the-art baselines across multiple benchmarks. It achieves stronger accuracy and robustness on continuous drug response tasks, while also generalizing effectively to discrete classification settings. Moreover, sensitivity analysis confirms the benefit of incorporating multiple topological scales, further supporting the importance of higher-order representations. Together, these results demonstrate that MolDr delivers reliable performance across heterogeneous pharmacogenomic scenarios and highlight the promise of topological modeling for advancing drug response prediction.

**Availability:** Source code freely available at https://github.com/CS-BIO/MolDr.

**Contact:** cshen@amss.ac.cn

**Supplementary information:** Supplementary data are available at *Bioinformatics* online.

**Key words:** Topological deep learning, Simplicial complex, Higher-order topology, Drug response prediction, Precision oncology.

## Methods

### Initialization of Simplex Features

After constructing the simplicial complexes, we assign initial features to simplices of different dimensions in order to parameterize them for subsequent learning. For 0-simplices, each vertex feature is initialized using pretrained embeddings obtained from Mol-GDL, which provide chemically meaningful representations of atomic environments:

$$X^{(0)} = \Phi_{\text{Mol-GDL}}(V) \in \mathbb{R}^{n_0 \times d_v}, \tag{1}$$

where $n_0$ denotes the number of vertices and $d_v$ is the embedding dimension.

For 1-simplices, each edge feature is derived from the corresponding bond length. Specifically, if $\ell(e)$ denotes the bond length of an edge $e$, then a multi-layer perceptron (MLP) is used to transform this scalar into a feature vector:

$$E_e^{(0)} = \phi_e(\ell(e)) \in \mathbb{R}^{d_e}. \tag{2}$$

For 2-simplices, each $\tau = (u, v, w)$ is characterized by the geometric property of its area. Given side lengths $a$, $b$, and $c$, the area of $\tau$ is computed using Heron's formula:

$$s = \frac{1}{2}(a + b + c), \quad \text{Area}(\tau) = \sqrt{s(s-a)(s-b)(s-c)}. \tag{3}$$

The computed area is then projected into a vector representation through another MLP:

$$T_\tau^{(0)} = \phi_t(\text{Area}(\tau)) \in \mathbb{R}^{d_t}. \tag{4}$$

Through this initialization scheme, simplices across different dimensions are consistently embedded into vector spaces

© The Author 2022. Published by Oxford University Press. All rights reserved. For permissions, please e-mail: journals.permissions@oup.com

that are suitable for neural message passing, thereby enabling the subsequent propagation layers to effectively integrate multi-dimensional structural information.

## Results

### Baselines

In order to comprehensively evaluate the performance of our proposed framework, we compared it against 15 representative baseline methods. All baselines were implemented according to their original publications and evaluated on the six benchmark datasets considered in this study, with hyperparameters set to their default values.

**SubCDR** (Liu and Zhang, 2023) introduces a subcomponent-guided deep learning framework, where drug molecules are decomposed into functional substructures and cell line profiles are divided into gene subsets. By modeling the interactions between drug and cell line "subcomponents," the method achieves improved prediction accuracy and highlights which molecular fragments or gene features drive drug response, thereby enhancing interpretability.

**GraphCDR** (Liu *et al.*, 2022) is a graph neural network framework enhanced with contrastive learning. It integrates multi-omics features of cancer cell lines, molecular graphs of drugs, and known drug–cell line responses into a unified graph representation. A contrastive learning task serves as a regularizer to improve the discriminative ability and generalization of the model. Experimental results demonstrate that GraphCDR outperforms state-of-the-art baselines under multiple settings and effectively alleviates data sparsity issues.

**PANCDR** (Kim *et al.*, 2024) proposes an adversarial learning-based framework that addresses the distribution shift between preclinical and clinical datasets. It consists of an adversarial module that reduces discrepancies between cell line and clinical data, and a prediction module for cancer drug response. By jointly training on preclinical and unlabeled clinical data, PANCDR achieves strong performance on external validation with TCGA and patient cohorts, demonstrating robustness and practical utility for precision medicine.

**MSDRP** (Zhao *et al.*, 2023) is a multi-source data-based deep learning model that integrates heterogeneous information about drugs and cell lines (e.g., chemical structure, targets, diseases, mutations) using a similarity network fusion (SNF) algorithm. An interaction module is further employed to capture pairwise relationships between drugs and cell lines. MSDRP shows superior performance compared with other baselines, particularly in de novo drug prediction tasks, and case studies confirm the rationality and interpretability of its similarity-based representations.

**MultiDRP** (Wang *et al.*, 2024) introduces a hierarchical attention framework that jointly captures external relationships among biomedical entities (via graph attention networks) and internal correlations within feature items (via self-attention). This design enables the model to learn multiscale relationships from both drug and cell line perspectives. MultiDRP achieves higher accuracy and robustness across diverse prediction scenarios, and in vitro validation further confirms the biological relevance of its predictions.

Simple Spectral Graph Convolution (**SSGC**) (Zhu and Koniusz, 2021) proposes a lightweight graph convolutional model that simplifies spectral filtering by decoupling feature transformation from propagation. By applying multiple steps of fixed graph diffusion followed by a linear transformation, SSGC reduces model complexity while preserving long-range information, making it efficient and competitive on various benchmark tasks.

**GraphGPS** (Rampášek *et al.*, 2022) is a general and scalable graph transformer architecture that unifies message passing neural networks (MPNNs) with transformer-style attention. It incorporates both local message passing for structural information and global attention for long-range dependencies, alongside positional encodings derived from graph Laplacians. This hybrid design enables GraphGPS to achieve state-of-the-art performance on large-scale graph learning benchmarks.

The Efficient Graph Convolution (**EGC**) (Tailor *et al.*, 2022) framework re-examines anisotropic GNNs and demonstrates that many existing variants can be unified under a shared formulation. EGC introduces an efficient scheme to combine multiple propagation operators with learned importance weights, thereby capturing directional and structural heterogeneity in graphs without incurring significant computational cost.

Graph Neural Networks with **ARMA** filters (Bianchi *et al.*, 2021) propose a novel convolutional layer inspired by auto-regressive moving average filters. Unlike polynomial-based spectral filters, ARMA filters provide a more flexible frequency response, capture global graph structures with fewer parameters, and are more robust to noise. This design allows ARMA-based GNNs to generalize better and outperform traditional polynomial filters across a range of tasks.

Anti-Symmetric Deep Graph Networks (**A-DGN**) (Gravina *et al.*, 2023) address the over-squashing problem in deep GNNs by formulating message passing through the lens of stable and non-dissipative ordinary differential equations. By enforcing anti-symmetric weight matrices, A-DGN preserves long-range dependencies and prevents gradient explosion or vanishing, enabling effective training of very deep graph networks.

**HGNN** (Hypergraph Neural Networks) (Feng *et al.*, 2019) extends graph neural networks to hypergraph structures, where each hyperedge can connect multiple nodes to model high-order relationships beyond pairwise interactions. It performs representation learning via a hyperedge convolution operation based on the hypergraph Laplacian, enabling node–edge–node message passing. This design allows HGNN to effectively capture complex and multi-modal data correlations, while traditional GCN can be viewed as a special case of HGNN when each hyperedge connects only two nodes.

**HGNN$^+$**(Gao *et al.*, 2022) extends the original HGNN framework by introducing a more flexible and general hypergraph modeling mechanism. Instead of directly concatenating multiple hypergraphs, it organizes high-order relationships into hyperedge groups and employs an adaptive fusion strategy to integrate multi-modal or multi-type information within a unified hypergraph. In addition, HGNN$^+$ adopts a spatial-domain hypergraph convolution with a vertex–hyperedge–vertex message passing scheme, enabling more expressive modeling of both low-order and high-order interactions.

**BScNets** (Bi-level Spectral Convolutional Networks) (Chen *et al.*, 2022) models graph data by combining spectral filtering at both node and subgraph levels. It leverages bi-level convolution to capture local node features and higher-level structural patterns simultaneously, enabling more expressive representation of complex graph structures.

**HiGCN** (Higher-order Graph Convolutional Network) (Huang *et al.*, 2024) models higher-order interactions by extending graph convolution to simplicial complexes. It introduces

a novel flower-petals representation and corresponding Laplacians to capture interactions across different simplex orders, and applies learnable spectral filters on each order to jointly model multi-scale higher-order structures.

**MolGT** (Chen *et al.*, 2025) is a graph Transformer-based molecular representation model that leverages self-attention with learnable bias to capture long-range dependencies in molecular graphs. It jointly models 2D topology and 3D geometry through a modality-shared Transformer and employs multimodal self-supervised pretraining to learn rich node- and graph-level representations.

## Datasets and Pre-processing

We benchmarked MolDr on six widely used pharmacogenomic resources: TGSA (Zhu *et al.*, 2022), CCLE (Barretina *et al.*, 2012), CTRP1 (Basu *et al.*, 2013), CTRP2 (Basu *et al.*, 2013), GDSC1 (Yang *et al.*, 2012; Iorio *et al.*, 2016; Garnett *et al.*, 2012), and GDSC2 (Yang *et al.*, 2012; Iorio *et al.*, 2016; Garnett *et al.*, 2012). To ensure comparability, all datasets were processed under a unified pipeline harmonizing molecular representations, cell line expression profiles, and drug response measurements. Response matrices were constructed from $IC_{50}$, AUC, or log-fold change values, followed by filtering to retain samples with complete information across modalities. For TGSA, drug–cell line pairs were provided with canonical SMILES and landmark gene expression. CCLE gene expression data (DepMap 24Q2) and PRISM drug responses were intersected, retaining 1,267 compounds and 221 cell lines with valid SMILES. CTRP1/2 datasets reported AUC values with molecular structures and expression data aligned to CCLE, yielding 203 drugs across 240 cell lines in CTRP1 and 545 drugs across 821 lines in CTRP2. GDSC1 and GDSC2 were obtained from the GDSC portal, reporting log-transformed $IC_{50}$ values with RNA-seq expression; after filtering, 312 drugs with 946 lines and 233 drugs with 944 lines remained, respectively.

For harmonization, $IC_{50}$ values were consistently transformed into `LN_IC50`, while AUC (CTRP) and LFC (CCLE) values were used in their original forms. Outliers were clipped at three standard deviations, and the top 1,000 most expressed genes were retained across datasets. Only samples with complete molecular, response, and expression profiles were included. The final dataset statistics are summarized in Table S1.

## Experimental setup

We propose MolDr, a topological deep learning framework that lifts molecular graphs into multiscale simplicial complexes and performs higher-order information propagation to predict drug response. Each molecule is represented as a family of Vietoris–Rips complexes constructed by bond-length-based filtration at cutoffs $\{2.0, 2.5, 3.0, 3.5, 4.0\}$ Å, where atoms, bonds, and three-vertex cliques are modeled as 0-, 1-, and 2-simplices, respectively. Within every scale, MolDr employs a two-layer propagation scheme: the first layer updates vertex (0-simplex) features by integrating signals from neighboring vertices together with incident 1- and 2-simplices; the second layer uses the updated vertices to refine 1- and 2-simplices and then feeds them back to vertices, forming a closed loop of higher-order information exchange. Initial features are assigned as follows: 0-simplices adopt Mol-GDL atom embeddings; 1-simplices are parameterized from bond lengths via an MLP; and 2-simplices are parameterized from their areas (Heron's formula) via another MLP. A permutation-invariant readout produces a molecular embedding per scale; multiscale embeddings are concatenated and projected by an MLP; the resulting molecular vector is fused with a cell-line gene-expression embedding and regressed to a drug response value with an MLP head trained by mean squared error. All methods share the same data protocol with an 8:1:1 split for training, validation, and testing. We report MAE (lower is better), PCC, and $R^2$ (both higher are better).

## Evaluation metrics

For regression tasks, three standard metrics were used: mean absolute error (MAE), Pearson correlation coefficient (PCC), and the coefficient of determination ($R^2$). Given $N$ samples with ground-truth responses $y_i$ and predicted values $\hat{y}_i$, MAE is defined as

$$\mathrm{MAE} = \frac{1}{N}\sum_{i=1}^{N} |y_i - \hat{y}_i| ,$$

which measures the average magnitude of prediction errors, with lower values indicating better accuracy. PCC evaluates the linear correlation between predictions and ground truth:

$$r = \frac{\sum_{i=1}^{N}(y_i - \bar{y})(\hat{y}_i - \bar{\hat{y}})}{\sqrt{\sum_{i=1}^{N}(y_i - \bar{y})^2}\sqrt{\sum_{i=1}^{N}(\hat{y}_i - \bar{\hat{y}})^2}},$$

where $\bar{y}$ and $\bar{\hat{y}}$ denote the sample means. PCC ranges from $-1$ to 1, with higher values indicating stronger linear association. The coefficient of determination $R^2$ quantifies how much variance in the data is explained by the model:

$$R^2 = 1 - \frac{\sum_{i=1}^{N}(y_i - \hat{y}_i)^2}{\sum_{i=1}^{N}(y_i - \bar{y})^2}.$$

An $R^2$ close to 1 suggests that the model predictions closely match the observed values.

For classification tasks, we employed the area under the ROC curve (AUC), accuracy, precision, and F1-score. AUC evaluates the quality of predicted probability scores $s_i \in [0, 1]$ by measuring the area under the receiver operating characteristic curve, where the true positive rate (TPR) and false positive rate (FPR) are given by

$$\mathrm{TPR} = \frac{\mathrm{TP}}{\mathrm{TP}+\mathrm{FN}}, \quad \mathrm{FPR} = \frac{\mathrm{FP}}{\mathrm{FP}+\mathrm{TN}}.$$

AUC can also be interpreted as the probability that a randomly chosen positive sample is ranked higher than a randomly chosen negative sample. Accuracy is defined as the fraction of correctly classified samples:

$$\mathrm{Accuracy} = \frac{\mathrm{TP}+\mathrm{TN}}{\mathrm{TP}+\mathrm{TN}+\mathrm{FP}+\mathrm{FN}}.$$

Precision measures the proportion of correctly predicted positive samples among all predicted positives:

$$\mathrm{Precision} = \frac{\mathrm{TP}}{\mathrm{TP}+\mathrm{FP}},$$

while recall (used in the computation of F1) is defined as

$$\mathrm{Recall} = \frac{\mathrm{TP}}{\mathrm{TP}+\mathrm{FN}}.$$

The F1-score is the harmonic mean of precision and recall:

$$\mathrm{F1} = \frac{2 \cdot \mathrm{Precision} \cdot \mathrm{Recall}}{\mathrm{Precision} + \mathrm{Recall}} = \frac{2\,\mathrm{TP}}{2\,\mathrm{TP}+\mathrm{FP}+\mathrm{FN}}.$$

This score balances the trade-off between precision and recall, and is especially informative when evaluating models on imbalanced datasets.

All metrics were computed on the held-out test sets, ensuring a fair comparison between methods. For multi-class or imbalanced scenarios, macro- or weighted-averaging schemes can be applied by averaging class-wise results either equally or in proportion to class sizes.

**Table S1.** Summary of frequently used mathematical symbols.

| Notation | Description |
|---|---|
| $X$ | Set of atomic coordinates |
| $\sigma^k$ | A $k$-simplex |
| $\tau$ | Face of a simplex |
| $\mathrm{Rips}_r(X)$ | Vietoris–Rips complex at cutoff $r$ |
| $A_{r,k}$ | Adjacency matrix of $k$-simplices |
| $D^U_{r,k}$ | Upper degree matrix |
| $D^L_{r,k}$ | Lower degree matrix |
| $H^{(l)}$ | Vertex representation after layer $l$ |
| $E$ | 1-simplex feature representation |
| $T$ | 2-simplex feature representation |
| $\rho(\cdot)$ | Readout (pooling) function |
| $\psi(\cdot)$ | Multilayer perceptron (MLP) |
| $z_{\mathrm{mol}}$ | Molecular representation |
| $z_{\mathrm{cell}}$ | Cell-line representation |
| $\hat{y}$ | Predicted drug response |

**Table S2.** Summary of drug response statistics across six pharmacogenomic datasets

| Dataset | Number of Drugs | Number of Cell Lines | Number of Drug Responses |
|---|---|---|---|
| TGSA | 170 | 580 | 82,833 |
| GDSC1 | 312 | 946 | 271,705 |
| GDSC2 | 233 | 944 | 198,457 |
| CCLE | 1267 | 221 | 278,597 |
| CTRP1 | 203 | 240 | 30,194 |
| CTRP2 | 545 | 821 | 364,774 |

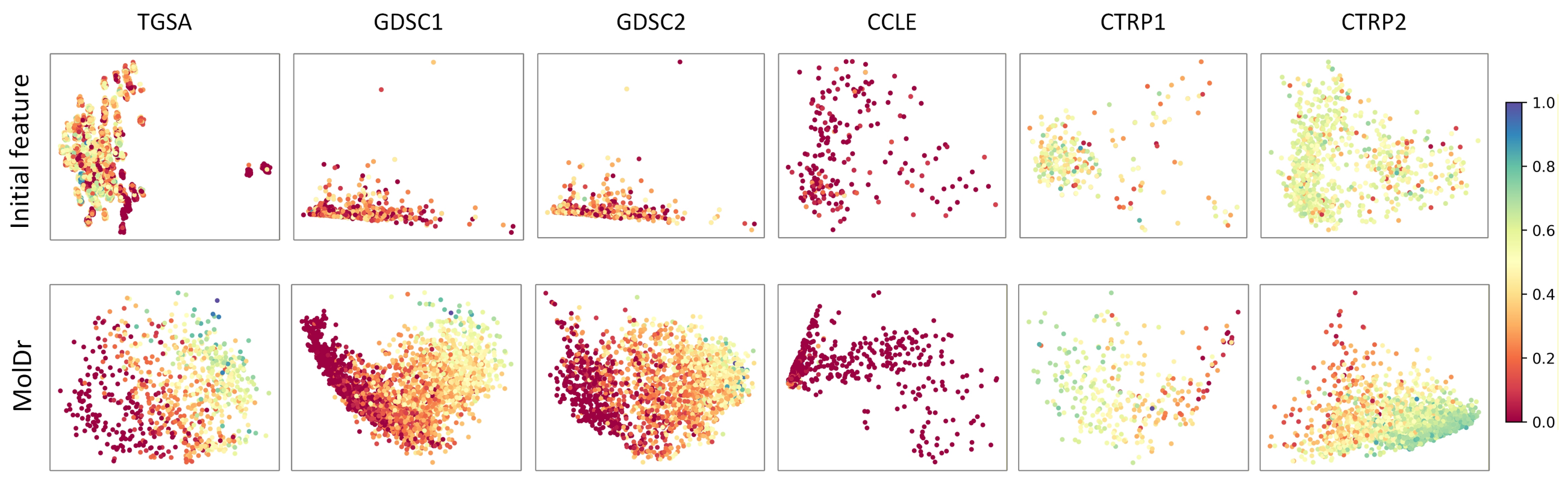


**Fig. S1. Visualization of drug–cell embeddings learned by MolDr on the test set using t-SNE.**

**Table S3.** Ablation study of the propagation depth.

| Dataset | Layer | MAE | PCC | $R^2$ | Dataset | Layer | MAE | PCC | $R^2$ |
|---|---|---|---|---|---|---|---|---|---|
| TGSA | 1-layer | 0.742 | 0.936 | 0.874 | GDSC1 | 1-layer | 0.752 | 0.924 | 0.854 |
| | 2-layer | 0.727 | 0.938 | 0.880 | | 2-layer | 0.722 | 0.929 | 0.863 |
| | 3-layer | 0.725 | 0.938 | 0.880 | | 3-layer | 0.732 | 0.927 | 0.860 |
| GDSC2 | 1-layer | 0.734 | 0.939 | 0.881 | CCLE | 1-layer | 0.477 | 0.863 | 0.743 |
| | 2-layer | 0.708 | 0.942 | 0.887 | | 2-layer | 0.469 | 0.878 | 0.768 |
| | 3-layer | 0.716 | 0.941 | 0.885 | | 3-layer | 0.478 | 0.864 | 0.745 |
| CTRP1 | 1-layer | 0.779 | 0.785 | 0.615 | CTRP2 | 1-layer | 0.836 | 0.892 | 0.795 |
| | 2-layer | 0.751 | 0.801 | 0.635 | | 2-layer | 0.795 | 0.903 | 0.814 |
| | 3-layer | 0.794 | 0.782 | 0.609 | | 3-layer | 0.794 | 0.902 | 0.812 |

**Table S4.** Ablation study of the cell-line embedding module.

| Dataset | Methods | MAE | PCC | $R^2$ | Dataset | Methods | MAE | PCC | $R^2$ |
|---|---|---|---|---|---|---|---|---|---|
| TGSA | Without cell embedding | 1.189 | 0.832 | 0.692 | GDSC1 | Without cell embedding | 1.033 | 0.853 | 0.728 |
| | Linear embedding | 0.764 | 0.932 | 0.868 | | Linear embedding | 0.736 | 0.936 | 0.857 |
| | MLP embedding (MolDr) | 0.727 | 0.938 | 0.880 | | MLP embedding (MolDr) | 0.722 | 0.929 | 0.863 |
| GDSC2 | Without cell embedding | 1.158 | 0.845 | 0.714 | CCLE | Without cell embedding | 0.497 | 0.846 | 0.714 |
| | Linear embedding | 0.735 | 0.939 | 0.881 | | Linear embedding | 0.476 | 0.864 | 0.745 |
| | MLP embedding (MolDr) | 0.708 | 0.942 | 0.887 | | MLP embedding (MolDr) | 0.469 | 0.878 | 0.768 |
| CTRP1 | Without cell embedding | 1.028 | 0.629 | 0.393 | CTRP2 | Without cell embedding | 1.172 | 0.766 | 0.586 |
| | Linear embedding | 0.739 | 0.810 | 0.646 | | Linear embedding | 0.818 | 0.895 | 0.801 |
| | MLP embedding (MolDr) | 0.751 | 0.801 | 0.635 | | MLP embedding (MolDr) | 0.795 | 0.903 | 0.814 |

**Table S5.** Ablation study of the multiscale fusion module.

| Dataset | Methods | MAE | PCC | $R^2$ | Dataset | Methods | MAE | PCC | $R^2$ |
|---|---|---|---|---|---|---|---|---|---|
| TGSA | Mean pooling | 0.725 | 0.939 | 0.880 | GDSC1 | Mean pooling | 0.716 | 0.929 | 0.863 |
| | Sum pooling | 0.716 | 0.940 | 0.882 | | Sum pooling | 0.718 | 0.929 | 0.863 |
| | Concatenation | 0.724 | 0.940 | 0.883 | | Concatenation | 0.717 | 0.929 | 0.864 |
| | Concatenation + MLP | 0.727 | 0.938 | 0.880 | | Concatenation + MLP | 0.722 | 0.929 | 0.863 |
| GDSC2 | Mean pooling | 0.710 | 0.942 | 0.887 | CCLE | Mean pooling | 0.470 | 0.867 | 0.752 |
| | Sum pooling | 0.713 | 0.942 | 0.886 | | Sum pooling | 0.479 | 0.865 | 0.748 |
| | Concatenation | 0.712 | 0.942 | 0.887 | | Concatenation | 0.473 | 0.867 | 0.752 |
| | Concatenation + MLP | 0.708 | 0.942 | 0.887 | | Concatenation + MLP | 0.469 | 0.878 | 0.768 |
| CTRP1 | Mean pooling | 0.769 | 0.789 | 0.618 | CTRP2 | Mean pooling | 0.795 | 0.901 | 0.812 |
| | Sum pooling | 0.758 | 0.797 | 0.632 | | Sum pooling | 0.798 | 0.902 | 0.812 |
| | Concatenation | 0.760 | 0.795 | 0.628 | | Concatenation | 0.788 | 0.903 | 0.814 |
| | Concatenation + MLP | 0.751 | 0.801 | 0.635 | | Concatenation + MLP | 0.795 | 0.903 | 0.814 |

**Table S6.** Cold-start performance under cell-line and drug split settings. Lower MAE and higher PCC/$R^2$ indicate better performance.

| Cell-line cold-start | | | | | Drug cold-start | | | | |
|---|---|---|---|---|---|---|---|---|---|
| Dataset | Method | MAE | PCC | $R^2$ | Dataset | Method | MAE | PCC | $R^2$ |
| TGSA | MultiDRP | 1.316 | 0.794 | 0.633 | TGSA | MultiDRP | 1.699 | 0.393 | 0.279 |
| | MolDr | **1.192** | **0.849** | **0.695** | | MolDr | **1.542** | **0.437** | **0.334** |
| GDSC1 | MultiDRP | 1.114 | 0.844 | 0.690 | GDSC1 | MultiDRP | 1.792 | 0.408 | 0.356 |
| | MolDr | **0.978** | **0.872** | **0.758** | | MolDr | **1.555** | **0.449** | **0.406** |
| GDSC2 | MultiDRP | 1.267 | 0.834 | 0.694 | GDSC2 | MultiDRP | 1.688 | 0.667 | 0.532 |
| | MolDr | **1.006** | **0.881** | **0.775** | | MolDr | **1.581** | **0.686** | **0.572** |
| CCLE | MultiDRP | 0.536 | 0.840 | 0.693 | CCLE | MultiDRP | 0.913 | 0.437 | 0.501 |
| | MolDr | **0.493** | **0.863** | **0.742** | | MolDr | **0.813** | **0.483** | **0.535** |
| CTRP1 | MultiDRP | 1.093 | 0.587 | 0.385 | CTRP1 | MultiDRP | 1.728 | 0.359 | 0.313 |
| | MolDr | **0.926** | **0.696** | **0.481** | | MolDr | **1.337** | **0.379** | **0.342** |
| CTRP2 | MultiDRP | 1.304 | 0.746 | 0.528 | CTRP2 | MultiDRP | 2.359 | 0.367 | 0.422 |
| | MolDr | **1.112** | **0.793** | **0.624** | | MolDr | **2.058** | **0.392** | **0.468** |

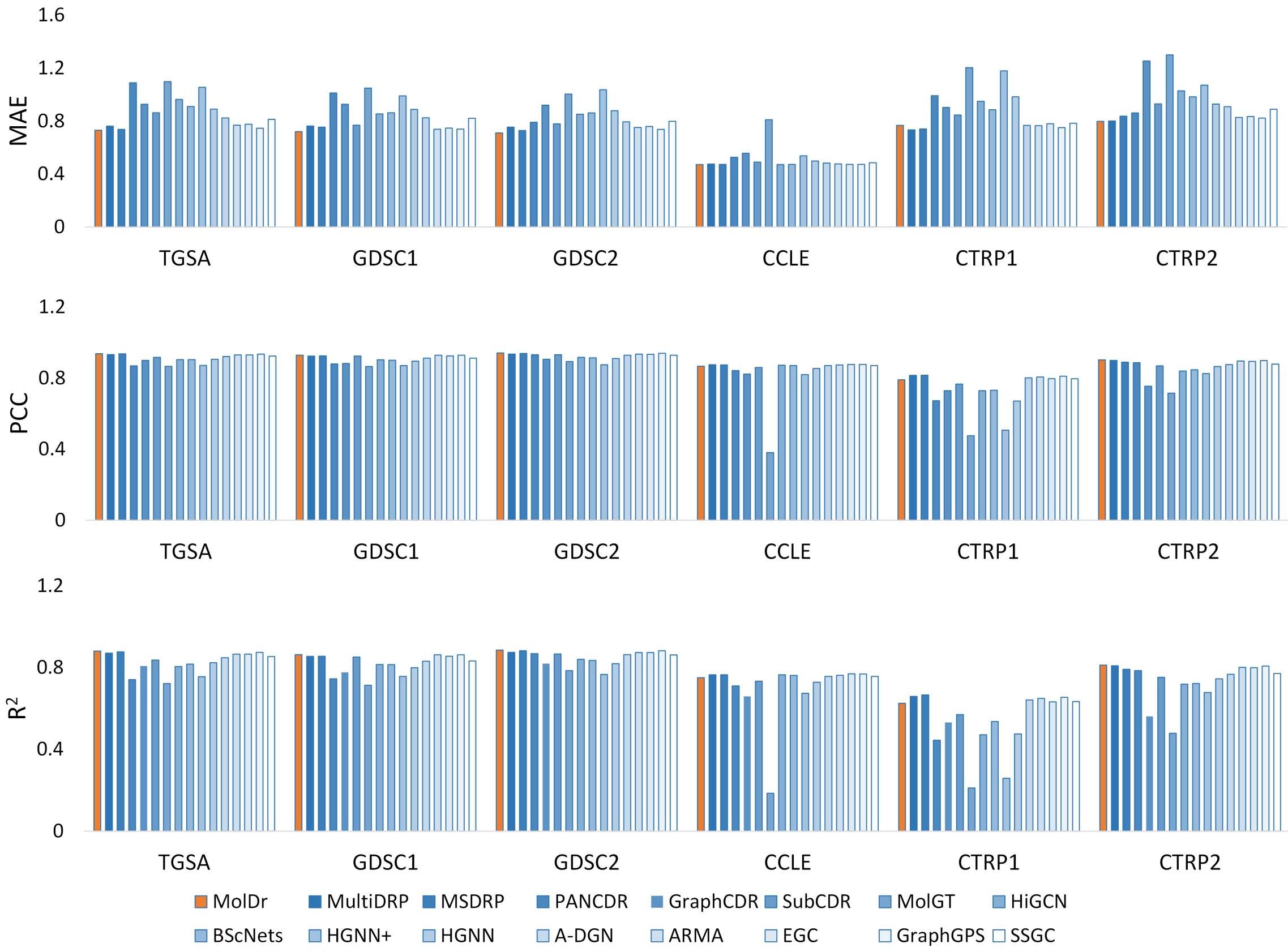


**Fig. S2. Performance under 20% random corruption of cell-line expression.** Grouped bar charts of MAE (lower is better), PCC, and $R^2$ (higher is better) across TGSA, GDSC1, GDSC2, CCLE, CTRP1, and CTRP2 for MolDr and baselines.

**Table S7.** Performance under 20% random corruption of cell-line expression. Lower MAE and higher PCC/$R^2$ are better. Best results in each row are in **bold**.

| Metric | Dataset | SSGC | GraphGPS | EGC | ARMA | A-DGN | HGNN | $HGNN^+$ | BScNets | HiGCN | MolGT | SubCDR | GraphCDR | PANCDR | MSDRP | MultiDRP | MolDr |
|---|---|---|---|---|---|---|---|---|---|---|---|---|---|---|---|---|---|
| MAE | TGSA | 0.812 | 0.745 | 0.776 | 0.769 | 0.824 | 0.890 | 1.055 | 0.910 | 0.963 | 1.097 | 0.862 | 0.927 | 1.089 | 0.736 | 0.761 | **0.731** |
| | GDSC1 | 0.821 | 0.740 | 0.747 | 0.738 | 0.825 | 0.877 | 0.991 | 0.862 | 0.854 | 1.048 | 0.768 | 0.927 | 1.012 | 0.754 | 0.763 | **0.720** |
| | GDSC2 | 0.798 | 0.737 | 0.759 | 0.752 | 0.795 | 0.878 | 1.037 | 0.861 | 0.852 | 1.004 | 0.778 | 0.920 | 0.790 | 0.728 | 0.754 | **0.710** |
| | CCLE | 0.485 | 0.472 | 0.473 | 0.476 | 0.483 | 0.498 | 0.537 | 0.472 | 0.471 | 0.810 | 0.489 | 0.557 | 0.527 | 0.473 | 0.475 | **0.471** |
| | CTRP1 | 0.783 | 0.750 | 0.780 | 0.765 | 0.767 | 0.982 | 1.179 | 0.886 | 0.949 | 1.203 | 0.846 | 0.902 | 0.992 | 0.740 | **0.733** | 0.767 |
| | CTRP2 | 0.890 | 0.822 | 0.834 | 0.827 | 0.908 | 0.928 | 1.070 | 0.983 | 1.028 | 1.300 | 0.930 | 1.253 | 0.861 | 0.837 | 0.800 | **0.798** |
| PCC | TGSA | 0.924 | 0.935 | 0.930 | 0.931 | 0.921 | 0.906 | 0.871 | 0.904 | 0.904 | 0.866 | 0.917 | 0.899 | 0.869 | 0.937 | 0.933 | **0.938** |
| | GDSC1 | 0.912 | 0.929 | 0.925 | 0.929 | 0.913 | 0.895 | 0.871 | 0.901 | 0.903 | 0.866 | 0.924 | 0.882 | 0.879 | 0.925 | 0.924 | **0.929** |
| | GDSC2 | 0.928 | 0.939 | 0.934 | 0.935 | 0.929 | 0.910 | 0.875 | 0.914 | 0.917 | 0.894 | 0.932 | 0.906 | 0.932 | 0.939 | 0.935 | **0.941** |
| | CCLE | 0.871 | **0.877** | **0.877** | 0.874 | 0.870 | 0.854 | 0.820 | 0.871 | 0.873 | 0.381 | 0.860 | 0.823 | 0.843 | 0.874 | 0.875 | 0.867 |
| | CTRP1 | 0.796 | 0.811 | 0.797 | 0.806 | 0.801 | 0.671 | 0.507 | 0.732 | 0.729 | 0.476 | 0.766 | 0.729 | 0.673 | **0.816** | 0.815 | 0.791 |
| | CTRP2 | 0.879 | 0.899 | 0.894 | 0.896 | 0.876 | 0.865 | 0.826 | 0.847 | 0.840 | 0.716 | 0.869 | 0.755 | 0.887 | 0.890 | 0.899 | **0.902** |
| $R^2$ | TGSA | 0.853 | 0.874 | 0.865 | 0.865 | 0.848 | 0.823 | 0.755 | 0.816 | 0.805 | 0.722 | 0.836 | 0.807 | 0.740 | 0.877 | 0.870 | **0.880** |
| | GDSC1 | 0.832 | 0.862 | 0.855 | 0.862 | 0.830 | 0.799 | 0.756 | 0.814 | 0.815 | 0.713 | 0.851 | 0.776 | 0.744 | 0.855 | 0.854 | **0.863** |
| | GDSC2 | 0.862 | 0.882 | 0.873 | 0.873 | 0.863 | 0.819 | 0.766 | 0.835 | 0.840 | 0.785 | 0.866 | 0.819 | 0.868 | 0.881 | 0.874 | **0.885** |
| | CCLE | 0.756 | 0.768 | **0.769** | 0.762 | 0.757 | 0.728 | 0.674 | 0.761 | 0.764 | 0.185 | 0.733 | 0.659 | 0.710 | 0.765 | 0.764 | 0.750 |
| | CTRP1 | 0.633 | 0.654 | 0.632 | 0.648 | 0.642 | 0.475 | 0.258 | 0.536 | 0.471 | 0.211 | 0.570 | 0.531 | 0.444 | **0.666** | 0.658 | 0.625 |
| | CTRP2 | 0.771 | 0.807 | 0.799 | 0.800 | 0.767 | 0.744 | 0.678 | 0.722 | 0.719 | 0.479 | 0.752 | 0.561 | 0.785 | 0.791 | 0.808 | **0.812** |

**Table S8.** Performance comparison on cancer-specific and pan-cancer settings.

| Cancer Type | Methods | Cancer-specific | | | Pan-cancer | | |
|---|---|---|---|---|---|---|---|
| | | MAE | PCC | $R^2$ | MAE | PCC | $R^2$ |
| BRCA | MiltiDRP | 0.8780 | 0.8947 | 0.7994 | 1.3236 | 0.7544 | 0.5228 |
| | MolDr | **0.8436** | **0.9015** | **0.8063** | **1.3005** | **0.7694** | **0.5688** |
| LUAD | MiltiDRP | 0.8448 | 0.9167 | 0.8285 | 1.2950 | 0.8101 | 0.6379 |
| | MolDr | **0.8427** | **0.9175** | **0.8347** | **1.2672** | **0.8150** | **0.6406** |
| COREAD | MiltiDRP | 0.8520 | 0.9109 | 0.8293 | 1.3208 | 0.8204 | 0.6068 |
| | MolDr | **0.8381** | **0.9158** | **0.8369** | **1.2854** | **0.8236** | **0.6141** |

**Table S9.** Sensitivity analysis of molecular conformer generation strategies.

| Dataset | Methods | MAE | PCC | $R^2$ | Dataset | Methods | MAE | PCC | $R^2$ |
|---|---|---|---|---|---|---|---|---|---|
| TGSA | Random embedding | 0.738 | 0.934 | 0.872 | GDSC1 | Random embedding | 0.719 | 0.929 | 0.862 |
| | ETKDG | 0.733 | 0.937 | 0.876 | | ETKDG | 0.720 | 0.929 | 0.863 |
| | ETKDG + MMFF | 0.730 | 0.937 | 0.878 | | ETKDG + MMFF | 0.726 | 0.928 | 0.860 |
| | Multi-conformer (lowest-energy) | 0.735 | 0.936 | 0.876 | | Multi-conformer (lowest-energy) | 0.729 | 0.927 | 0.859 |
| GDSC2 | Random embedding | 0.723 | 0.939 | 0.884 | CCLE | Random embedding | 0.481 | 0.864 | 0.746 |
| | ETKDG | 0.721 | 0.940 | 0.886 | | ETKDG | 0.479 | 0.864 | 0.747 |
| | ETKDG + MMFF | 0.717 | 0.940 | 0.885 | | ETKDG + MMFF | 0.475 | 0.861 | 0.753 |
| | Multi-conformer (lowest-energy) | 0.725 | 0.939 | 0.884 | | Multi-conformer (lowest-energy) | 0.478 | 0.859 | 0.751 |
| CTRP1 | Random embedding | 0.773 | 0.792 | 0.616 | CTRP2 | Random embedding | 0.798 | 0.901 | 0.811 |
| | ETKDG | 0.770 | 0.790 | 0.621 | | ETKDG | 0.799 | 0.901 | 0.811 |
| | ETKDG + MMFF | 0.756 | 0.800 | 0.626 | | ETKDG + MMFF | 0.798 | 0.901 | 0.811 |
| | Multi-conformer (lowest-energy) | 0.770 | 0.798 | 0.625 | | Multi-conformer (lowest-energy) | 0.799 | 0.901 | 0.810 |

**Table S10. Sensitivity analysis of molecular conformer uncertainty.** For each dataset, 100 molecules were randomly selected, and 20 conformers were generated for each molecule using ETKDG followed by MMFF/UFF optimization. The reported values are averaged across five filtration cutoffs (2.0–4.0 Å). Lower CV indicates higher stability, while higher geometry similarity indicates greater consistency across conformers.

| Dataset | Geometry Similarity | Edge CV | Triangle CV | Edge Length CV | Triangle Area CV |
|---|---|---|---|---|---|
| TGSA | 0.930 | 0.0104 | 0.0242 | 0.0037 | 0.0103 |
| GDSC1 | 0.931 | 0.0110 | 0.0283 | 0.0040 | 0.0139 |
| GDSC2 | 0.931 | 0.0110 | 0.0283 | 0.0040 | 0.0139 |
| CCLE | 0.918 | 0.0115 | 0.0255 | 0.0041 | 0.0117 |
| CTRP1 | 0.928 | 0.0109 | 0.0241 | 0.0039 | 0.0110 |
| CTRP2 | 0.884 | 0.0142 | 0.0313 | 0.0041 | 0.0126 |

**Table S11.** Performance under different multiscale filtration cutoff windows.

| Dataset | Cutoff values | MAE | PCC | $R^2$ | Dataset | Cutoff values | MAE | PCC | $R^2$ |
|---|---|---|---|---|---|---|---|---|---|
| TGSA | {1.5, 2.0, 2.5, 3.0}Å | 0.733 | 0.936 | 0.877 | CTRP1 | {1.5, 2.0, 2.5, 3.0}Å | 0.765 | 0.795 | 0.625 |
| | {2.0, 2.5, 3.0, 3.5}Å | 0.727 | 0.938 | 0.880 | | {2.0, 2.5, 3.0, 3.5}Å | 0.751 | 0.801 | 0.635 |
| | {2.5, 3.0, 3.5, 4.0}Å | 0.727 | 0.938 | 0.878 | | {2.5, 3.0, 3.5, 4.0}Å | 0.755 | 0.799 | 0.634 |
| | {3.0, 3.5, 4.0, 4.5}Å | 0.727 | 0.937 | 0.878 | | {3.0, 3.5, 4.0, 4.5}Å | 0.757 | 0.797 | 0.631 |

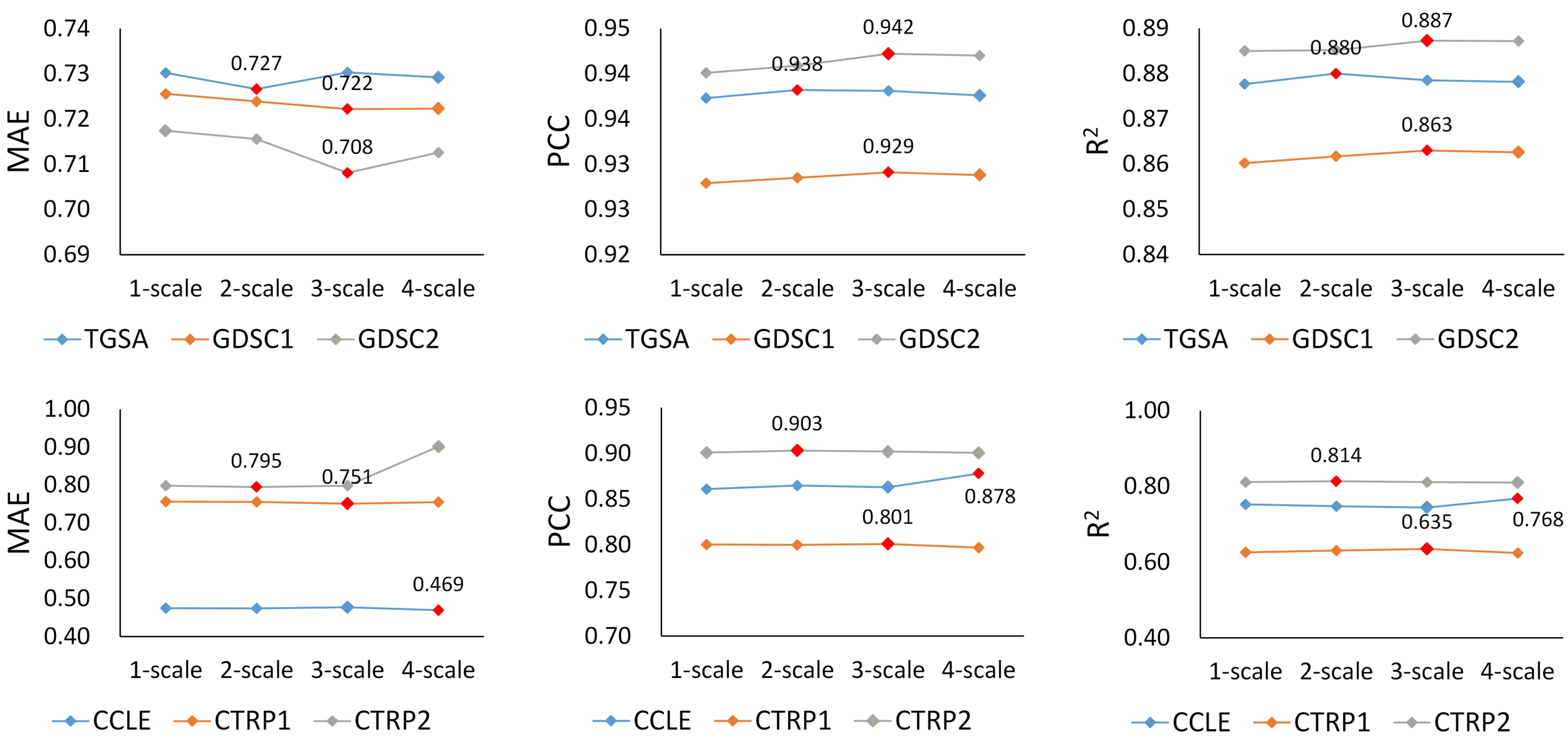


**Fig. S3. Performance comparison under different scale settings**

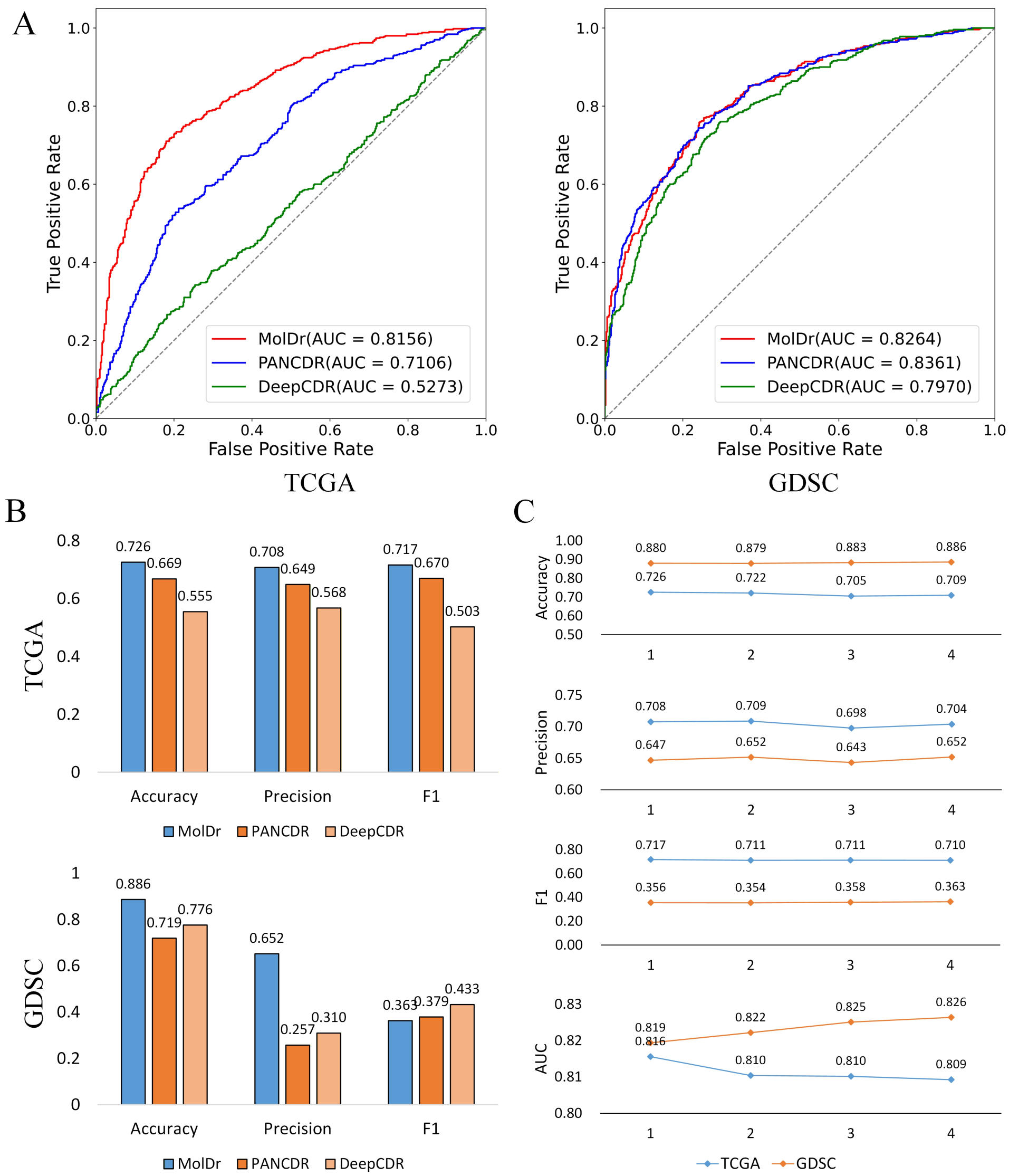


**Fig. S4. Performance of MolDr on discrete drug response datasets (TCGA and GDSC).** (A) ROC curves comparing MolDr with PANCDR and DeepCDR. (B) Accuracy, precision, and F1-score comparisons across methods. (C) Parameter sensitivity analysis under different numbers of filtration intervals.

## References


Barretina, J. *et al.* (2012). The cancer cell line encyclopedia enables predictive modelling of anticancer drug sensitivity. *Nature*, **483**(7391), 603–607.

Basu, A. *et al.* (2013). An interactive resource to identify cancer genetic and lineage dependencies targeted by small molecules. *Cell*, **154**(5), 1151–1161.

Bianchi, F. M. *et al.* (2021). Graph neural networks with convolutional arma filters. *IEEE transactions on pattern analysis and machine intelligence*, **44**(7), 3496–3507.

Chen, R. *et al.* (2025). Pretraining graph transformer for molecular representation with fusion of multimodal information. *Information Fusion*, **115**, 102784.

Chen, Y. *et al.* (2022). BScNets: Block simplicial complex neural networks. In *Proceedings of the AAAI Conference on Artificial Intelligence*, volume 36, pages 6333–6341.

Feng, Y. *et al.* (2019). Hypergraph neural networks. In *Proceedings of the AAAI Conference on Artificial Intelligence*, volume 33, pages 3558–3565.

Gao, Y. *et al.* (2022). HGNN+: General hypergraph neural networks. *IEEE Transactions on Pattern Analysis and Machine Intelligence*, **45**(3), 3181–3199.

Garnett, M. J. *et al.* (2012). Systematic identification of genomic markers of drug sensitivity in cancer cells. *Nature*, **483**(7391), 570–575.

Gravina, A. *et al.* (2023). Anti-symmetric DGN: a stable architecture for deep graph networks. In *The Eleventh International Conference on Learning Representations*.

Huang, Y. *et al.* (2024). Higher-order graph convolutional network with flower-petals laplacians on simplicial complexes. In *Proceedings of the AAAI Conference on Artificial Intelligence*, volume 38, pages 12653–12661.

Iorio, F. *et al.* (2016). A landscape of pharmacogenomic interactions in cancer. *Cell*, **166**(3), 740–754.

Kim, J. *et al.* (2024). Pancdr: precise medicine prediction using an adversarial network for cancer drug response. *Briefings in Bioinformatics*, **25**(2), bbae088.

Liu, X. and Zhang, W. (2023). A subcomponent-guided deep learning method for interpretable cancer drug response prediction. *PLOS Computational Biology*, **19**(8), e1011382.

Liu, X. *et al.* (2022). Graphcdr: a graph neural network method with contrastive learning for cancer drug response prediction. *Briefings in Bioinformatics*, **23**(1), bbab457.

Rampášek, L. *et al.* (2022). Recipe for a general, powerful, scalable graph transformer. *Advances in Neural Information Processing Systems*, **35**, 14501–14515.

Tailor, S. A. *et al.* (2022). Adaptive filters for low-latency and memory-efficient graph neural networks. In *International Conference on Learning Representations*.

Wang, X. *et al.* (2024). A hierarchical attention network integrating multi-scale relationship for drug response prediction. *Information Fusion*, **110**, 102485.

Yang, W. *et al.* (2012). Genomics of drug sensitivity in cancer (gdsc): a resource for therapeutic biomarker discovery in cancer cells. *Nucleic acids research*, **41**(D1), D955–D961.

Zhao, H. *et al.* (2023). Msdrp: a deep learning model based on multisource data for predicting drug response. *Bioinformatics*, **39**(9), btad514.

Zhu, H. and Koniusz, P. (2021). Simple spectral graph convolution. In *International conference on learning representations*.

Zhu, Y. *et al.* (2022). Tgsa: protein–protein association-based twin graph neural networks for drug response prediction with similarity augmentation. *Bioinformatics*, **38**(2), 461–468.
